# Cancer Treatment with Immune Checkpoint Inhibition in Solid Organ Transplant Recipients in Switzerland

**DOI:** 10.3390/cancers18121918

**Published:** 2026-06-12

**Authors:** Rahel Looser, Günther F. L. Hofbauer, Dela Golshayan, Mirjam C. Nägeli, on behalf of the Swiss Transplant Cohort Study

**Affiliations:** 1 Department of Dermatology, University Hospital Zurich, 8091 Zurich, Switzerland; gfl@hofbauer.ch (G.F.L.H.); mirjam.naegeli@usz.ch (M.C.N.); 2 Faculty of Medicine, University of Zurich, 8032 Zurich, Switzerland; 3 allderm, Allergology and Dermatology, Guyer-Zeller-Strasse 10, 8620 Wetzikon, Switzerland; 4 Transplantation Centre and Transplantation Immunopathology Laboratory, Department of Medicine, Lausanne University Hospital, University of Lausanne, 1011 Lausanne, Switzerland; dela.golshayan@chuv.ch

**Keywords:** ICI, checkpoint inhibitor, checkpoint inhibition, solid organ transplant recipients, transplant patients, advanced malignancies

## Abstract

**Background/Objectives**: There is limited data on treatment outcomes under immune checkpoint inhibitor (ICI) administration in solid organ transplant recipients (sOTRs). This study aims to evaluate cancer outcome and allograft rejection risk in sOTRs receiving ICI. **Methods**: This is a retrospective multicenter study. The data had been collected within the Swiss Transplant Cohort Study (STCS) database. We searched for matching individuals from May 2008 up to the end of 2024. The primary outcomes were treatment response and survival; the secondary outcome was allograft rejection. Additional analyses included associated factors such as tumor, transplant, and treatment characteristics. **Results**: We identified ten patients, six of whom received a kidney allograft, while the remaining four received a liver, lung, pancreas, or combined kidney–pancreas transplant. Treatment response was achieved in half of the sOTRs, with a complete response (CR) in three and prolonged stable disease (SD) in two patients. CR was achieved in all three patients after only a few infusions. At the time of data analysis, four out of ten patients were still alive. Graft rejection occurred in six out of ten cases, five of which occurred after the first cycle of ICI administration. **Conclusions**: Data is limited and definitive conclusions from this study cannot be drawn given the limited sample size. However, ICI displays promising effects on cancer outcomes in sOTRs with advanced malignancies. The study’s findings demonstrate an overall response in half of sOTRs, but with graft rejection occurring in a similar number of patients. We propose initiating immunotherapy as early as possible, given the promising results, particularly in patients with kidney transplants. We further suggest that in sOTRs, a few ICI infusions could potentially be a cautious option, pending further evidence.

## Simple Summary

Organ transplant recipients must take medication to weaken their immune system, which increases their risk of developing aggressive cancers. New cancer treatments, such as immunotherapy, activate the immune system to fight cancer but may also cause organ rejection. In this study, we examine transplant patients in Switzerland who had advanced malignancies and were treated with immunotherapy. Our goal was to evaluate the treatment outcome. We identified ten patients, three of whom became cancer-free, and two whose cancer stopped growing for several months. Four out of ten patients were still alive at the time of data analysis. Two of them were cancer-free. However, six patients developed organ rejection. Overall, immunotherapy is promising, as more than half of the patients showed a response. We suggest starting immunotherapy as early as possible, and a few infusions may be sufficient. Each case should be carefully discussed by doctors together with the patient.

## 1. Introduction

There is limited data on cancer outcomes under immune checkpoint inhibitor (ICI) administration in solid organ transplant recipients (sOTRs). ICI has been used only reluctantly due to fear of graft rejection [1], although malignancy is greatly increased in sOTRs [2,3]. This greater risk is multifactorial and associated with oncogenic viral infection, altered T-cell immunity, immunosuppressants and immunosuppression at large [4]. In oncology, checkpoint inhibition with ICI has quickly become one of the most promising emerging approaches. The melanoma-specific survival rate with ICI combination therapy in patients with advanced, previously untreated melanoma was 52% after ten years [5]. In patients with advanced cutaneous squamous cell carcinoma (SCC), therapy response was accomplished with ICI in half of the study population [6,7]. ICIs promote T-cell activation by blocking, e.g., programmed cell death protein 1 (PD-1), programmed cell death ligand 1 (PD-L1), or cytotoxic T-lymphocyte-associated protein 4 (CTLA-4) [8]. PD-L1 on neoplastic cells binds to PD-1 on T-cells and other immune cells to inhibit their activity. CTLA-4 suppresses the T-cell co-stimulation and activation [9]. These processes lead to an immune response not only against malignant cells but also against donor alloantigens of sOTRs [10,11]. In the literature, allograft rejection under ICI administration was found in 0% to 54% of the patients [12,13,14]. Nonetheless, ICI administration in sOTRs appears to be successful for cancer control in a supervised setting. The overall response rate ranged from 20% to 57% [12,13,14,15,16]. Potential confounders like patients’ characteristics, type of cancer and allograft, therapy regime and other factors may have an impact on treatment success and graft rejection.

This study aims to analyze sOTRs in Switzerland with a history of ICI administration due to advanced malignancies, collected within the Swiss Transplant Cohort Study (STCS) from 2008 up to the end of 2024. Treatment success and safety are the study’s main outcomes.

## 2. Materials and Methods

The trial design is a retrospective multicenter study. The data had been collected in Switzerland’s six transplantation centers (Cantonal Hospital St. Gallen, University Hospital Basel, Bern, Geneva, Lausanne and Zurich), coded and stored within the STCS database since May 2008. We searched the database for sOTRs with advanced neoplasms (unresectable primary tumor or metastases) treated with ICI. The only exclusion criterion was a documented objection to the subsequent use of personal health data. The primary outcome was the response to cancer treatment, classified as progressive disease (PD), stable disease (SD), partial response (PR), and complete response (CR). If data were available, analyses of time to relapse, progression-free survival (PFS) and cause of death were captured. The secondary outcome was the evaluation of allograft rejection. Associated factors were analyzed, including patient characteristics, type of cancer and allograft, ICI therapy regimen, and concomitant immunosuppression. Descriptive statistics were performed to summarize the data. For continuous variables, the arithmetic mean was calculated. No inferential statistical tests were performed, given the limited sample size. All results are reported descriptively.

## 3. Results

**Figure A1:**
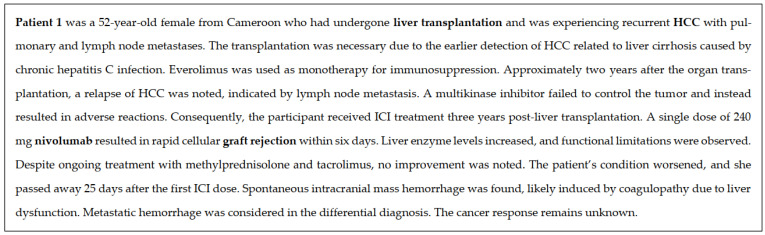
Summarized patients’ histories: Patient 1. Abbreviations: ICI, immune checkpoint inhibitor; HCC, hepatocellular carcinoma.

**Figure A2:**
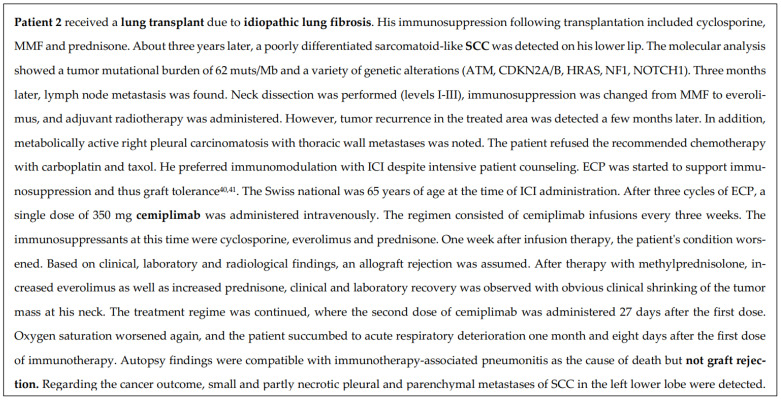
Summarized patients’ histories: Patient 2 [40,41]. Abbreviations: ECP, extracorporeal photopheresis; ICI, immune checkpoint inhibitor; MMF, mycophenolate mofetil; SCC, cutaneous squamous cell carcinoma.

**Figure A3:**
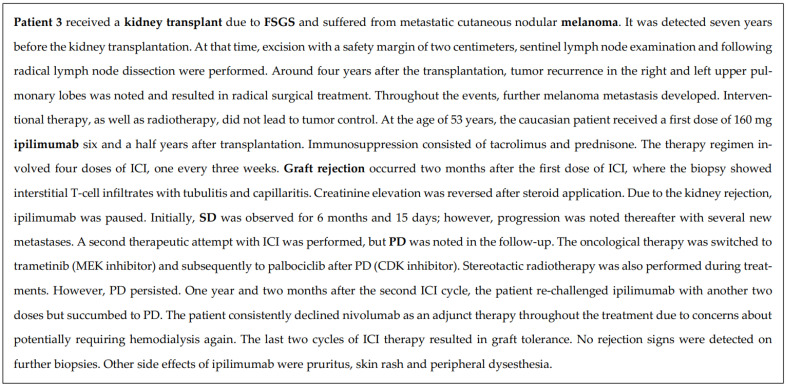
Summarized patients’ histories: Patient 3. Abbreviations: ICI, immune checkpoint inhibitor; FSGS, primary focal segmental glomerulosclerosis; PD, progressive disease; SD, stable disease.

**Figure A4:**
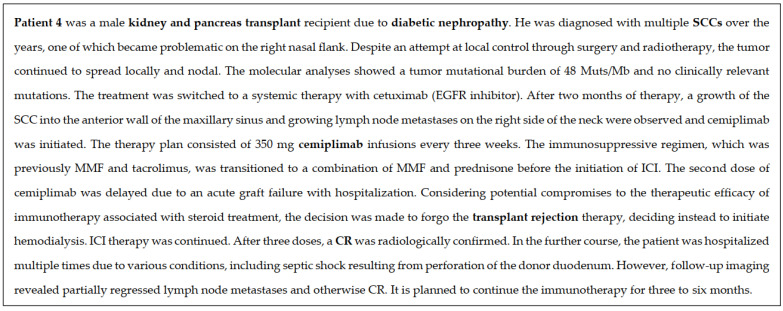
Summarized patients’ histories: Patient 4. Abbreviations: CR, complete response; ICI, immune checkpoint inhibitor; MMF, mycophenolate mofetil; SCC, cutaneous squamous cell carcinoma.

**Figure A5:**
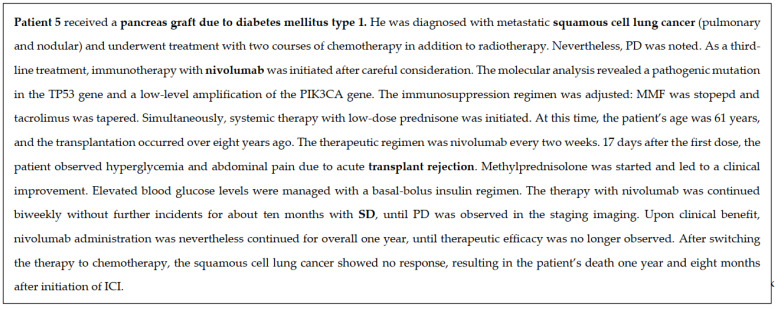
Summarized patients’ histories: Patient 5. Abbreviations: ICI, immune checkpoint inhibitor; MMF, mycophenolate mofetil; PD, progressive disease; SD, stable disease.

**Figure A6:**
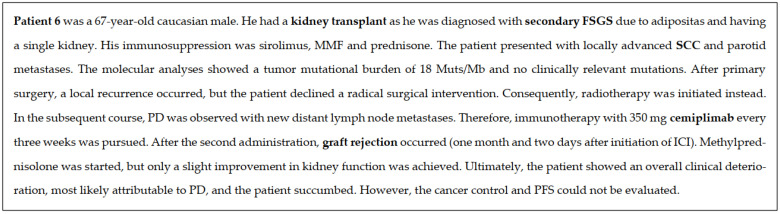
Summarized patients’ histories: Patient 6. Abbreviations: ICI, immune checkpoint inhibitor; FSGS, primary focal segmental glomerulosclerosis; MMF, mycophenolate mofetil; PD, progressive disease; PFS, progression-free survival; SCC, cutaneous squamous cell carcinoma.

**Figure A7:**
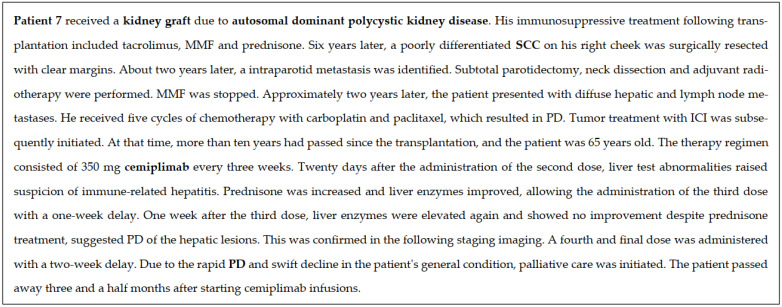
Summarized patients’ histories: Patient 7. Abbreviations: ICI, immune checkpoint inhibitor; MMF, mycophenolate mofetil; PD, progressive disease; SCC, cutaneous squamous cell carcinoma.

**Figure A8:**
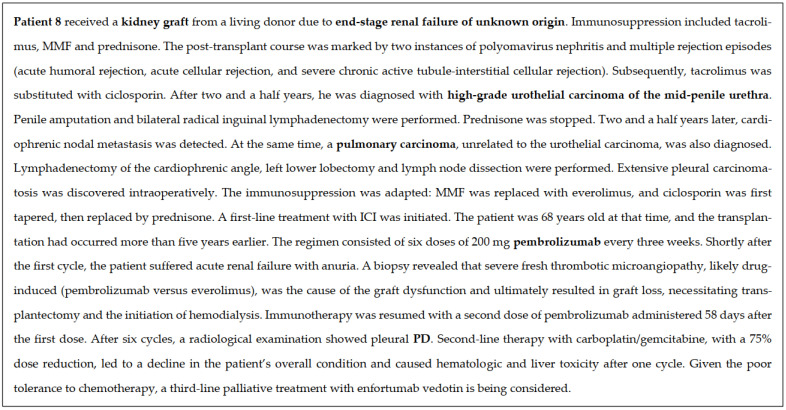
Summarized patients’ histories: Patient 8. Abbreviations: ICI, immune checkpoint inhibitor; MMF, mycophenolate mofetil; PD, progressive disease.

**Figure A9:**
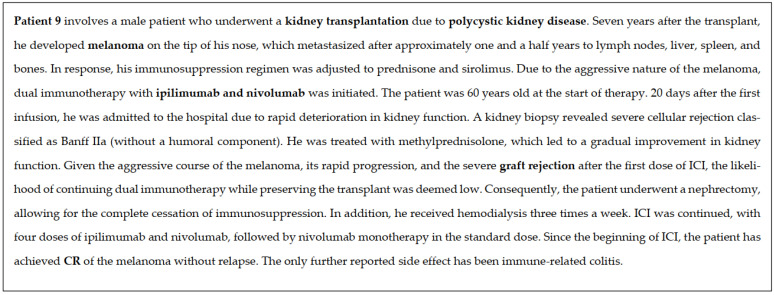
Summarized patients’ histories: Patient 9. Abbreviations: CR, complete response; ICI, immune checkpoint inhibitor; MMF, mycophenolate mofetil.

**Figure A10:**
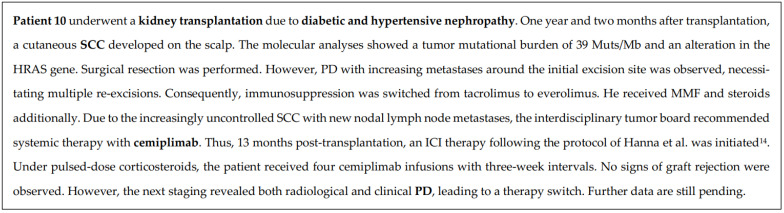
Summarized patients’ histories: Patient 10 [14]. Abbreviations: ICI, immune checkpoint inhibitor; MMF, mycophenolate mofetil; PD, progressive disease; SCC, cutaneous squamous cell carcinoma.

Within the STCS database, we identified ten patients matching the inclusion criteria. The demographics and patient characteristics are summarized in Table 1, and the patients’ histories are displayed in Figure A1, Figure A2, Figure A3, Figure A4, Figure A5, Figure A6, Figure A7, Figure A8, Figure A9 and Figure A10. Nine of the ten participants were male. The mean age at the point of ICI administration was 64 years. The cases include a liver, a lung, a pancreas, a simultaneous kidney–pancreas transplant, and six kidney graft recipients. Five patients had metastatic cutaneous SCC, two patients had metastatic melanoma (MM), and the remaining patients suffered from advanced hepatocellular carcinoma (HCC), squamous cell lung cancer, and high-grade urothelial carcinoma of the mid-penile urethra. Regarding immunosuppression, eight out of the ten patients received combination therapy with steroids at the start of immunotherapy. Five patients received a dual combination of mammalian target of rapamycin (mTOR) inhibitors and prednisone, and three others were given an additional third immunosuppressant (mycophenolate mofetil (MMF) or cyclosporine).

Patients with metastatic SCC received ICI therapy with cemiplimab. The patient with HCC and the one with metastatic squamous cell lung cancer were treated with nivolumab monotherapy. The two patients with MM received ipilimumab, one as monotherapy and the other with an ipilimumab/nivolumab combination, followed by nivolumab monotherapy. The treatment regimen is listed for each patient in Table 1.

### 3.1. Primary Outcome

The primary outcome is summarized in Table 2. Three out of the ten patients achieved a CR under immunotherapy. One of them showed CR after two, and one after three infusions. The third patient showed CR in the first staging after ICI initiation. Two patients reached SD but subsequently progressed to PD. The mean progression-free interval of these two patients was seven months and 28 days. Three patients showed PD in the follow-up imaging after a mean of three months and nine days. The remaining two cases could not be evaluated for tumor response due to rapid death and the lack of autopsy or imaging after the initiation of ICI.

At the time of data analysis, four of the patients were still alive, while six patients had passed away. Four of these deaths were due to PD. A lung transplant recipient died because of immune-related pneumonitis, leading to severe respiratory insufficiency, and one patient passed away either due to graft dysfunction or PD (unknown). The mean duration from ICI administration to death was ten months for the patients who passed away.

### 3.2. Secondary Outcome

The secondary outcome is summarized in Table 3. Graft loss was observed in six patients. Four of these cases were attributable to graft rejection because of ICI. Of the remaining two, one was immune-mediated and the other was most likely secondary to drug-induced thrombotic microangiopathy (everolimus vs. ICI). Allograft rejection occurred in six out of ten cases. Four of them were treated with PD-1 inhibition, one with anti-CTLA-4 monotherapy, and the remaining with both as a combination therapy. In all but one patient, graft rejection was observed after the first dose of immunotherapy.

The mean period between ICI administration and allograft rejection was 26 days. If treatment was initiated, high-dose methylprednisolone was the primary therapy. Three cases showed a clinical response to treatment. In two patients, organ function failed to recover, whereas in another patient, active therapy was not performed to minimize steroid usage, and dialysis was initiated.

## 4. Discussion

### 4.1. Primary Outcome: Treatment Response

Our study showed a CR in three patients and SD for several months in two patients under ICI. Overall, cancer control was achieved in half of the study population. These results suggest that immunotherapy with ICI shows promising outcomes even in sOTRs. CR was achieved in all three patients after only a few infusions.

In sOTRs with advanced **SCC**, cancer response was accomplished with anti-PD-1 cemiplimab in approximately half of the study population [6,7,14]. There are other case reports demonstrating excellent SCC control under PD-1 inhibitors [17,18]. An advantage in overall survival is described [15]. Consistent with previous reports, our data showed a CR under PD-1 inhibitor in half of the study population suffering from advanced SCC.

The **melanoma**-specific survival rate to ICI combination therapy was up to 52% after a follow-up of ten years in the non-sOTRs population [5]. Studies suggest that therapeutic success can also be achieved in the sOTRs population in more than half of patients, with combination therapy showing the most promising results [15,16,19,20,21]. In our study, the patient treated with ipilimumab alone maintained SD for several months, while the patient receiving combination therapy achieved a CR.

According to Pinter et al., response rates of **HCC** to ICI monotherapy ranged from 15% to 23% and increased to approximately 30% after combination immunotherapy [22]. A wide variability in treatment response has been observed in other reports on HCC cases managed with PD-1 inhibitors, with outcomes ranging from poor to excellent [12,23,24,25,26,27,28,29].

### 4.2. Secondary Outcome: Side Effects and Influencing Factors on Allograft Rejection

Graft rejection occurred in six out of ten cases. Concerning the influence of ICI type on graft rejection, we found that the patients who showed graft tolerance were treated with anti-PD-1 inhibitors. In contrast, other studies suggest that the prescription of PD-1 inhibitors has a higher risk of allograft rejection compared to anti-CTLA-4 application [10,12,13,30]. The underlying mechanism remains unclear, but the different pathways of ICI action might be an explanation. The CTLA-4 pathway is found to be essential for transplant tolerance induction, but less for the maintenance of already existing graft tolerance [31]. The PD-1 pathway, on the other hand, is important for long-term transplant survival [32]. In Switzerland, only two patients have been treated with anti-CTLA-4 mono- or combination therapy to date, while the remaining eight patients received anti-PD-1 monotherapy. Therefore, no conclusions can be drawn regarding CTLA-4 inhibition and the risk of graft rejection, given the small study population.

Regarding the influence of the graft type on its risk for rejection, several studies indicate a greater risk of graft rejection in kidney-transplant patients than in liver-transplant patients under ICI treatment [10,12,13,15]. This correlates with the overall risk for rejection in sOTRs, which is, in general, higher in kidney and lung than in liver transplant recipients [33]. Two of the patients in our database who exhibited graft tolerance are both kidney transplant recipients. However, kidney transplant recipients are also overrepresented in our study population (seven out of ten) as they can get hemodialysis in case of a graft rejection.

Data about the influence of concomitant immunosuppression on the graft rejection rate is rarely described. Studies suggest that a higher number of immunosuppressant drugs may provide graft tolerance. It is also postulated that transplant tolerance may be achieved by maintaining baseline immunosuppression during ICI treatment without affecting the therapeutic outcome [15,34]. Furthermore, concomitant immunosuppression with an mTOR inhibitor turned out to be prophylactic for acute rejection, with prolonged overall and especially rejection-free graft survival [14,15]. Nevertheless, mTOR inhibitors appear to be less well tolerated and are associated with a higher incidence of adverse events compared to other immunosuppressive therapies [35]. Three of the four patients in the Swiss database who exhibited graft tolerance received an mTOR inhibitor. Unfortunately, higher blood levels of steroids are associated with an inferior cancer outcome by impairing the effectiveness of anti-PD-1 therapy significantly [36].

Regarding other side effects of ICI application than graft rejection, CTLA-4 inhibitors seem to be generally less tolerable [10].

In the study population, 9 out of 10 cases were male. In published statistics, male patients are more likely to obtain a transplant with an overrepresentation of 65% [37]. Nevertheless, the generalizability of the study’s findings to female sOTRs remains limited and unclear.

## 5. Conclusions

Overall, the ICI application displays promising effects on cancer outcomes in patients with advanced malignancies. Data is limited and definitive conclusions from this study cannot be drawn given the limited sample size.

Recently published data showed tumor response to ICI therapy in sOTRs without loss of graft [34,38]. SCC and MM seem to be more responsive than recurrent HCC in sOTRs, where nivolumab remains a second-line treatment [12,25,26,27,28,29]. Based on our observations and the currently available literature, we propose initiating immunotherapy as early as possible, especially in patients with MM and advanced SCC. In the sOTRs population, advanced neoplasm leads to rapid death, so the therapy with the highest response rate should be used. Patients with kidney transplants must be considered as a separate group, as these patients have the option of hemodialysis after organ rejection, while transplant loss is fatal for other sOTRs. We further suggest that in sOTRs, dependent on their graft for survival, only a few ICI infusions could potentially be a considerate option, as some data suggest in the non-sOTRs population [39] and in this study population. However, given the extremely limited sample size within each subgroup in the current study, this remains a clinical hypothesis requiring further investigation. Each case should be individually and interdisciplinarily discussed at the tumor board and with the patient. However, making evidence-based recommendations is extremely difficult; further analysis of published data may provide more guidance for management of ICI in sOTRs.

## Figures and Tables

**Table 1:** Demographics and patient characteristics.

Patient	1	2	3	4	5	6	7	8	9	10
Gender	Female	Male	Male	Male	Male	Male	Male	Male	Male	Male
Age *	52 y	65 y	63 y	68 y	61 y	67 y	64 y	67 y	60 y	74 y
Weight *	61 kg	81 kg	53 kg	66 kg	65 kg	84 kg	79 kg	UNK	87 kg	77 kg
Ethnicity	Cameroonian	Caucasian	Caucasian	Caucasian	Caucasian	Caucasian	Caucasian	Caucasian	Caucasian	Caucasian
Allograft	Liver	Lung	Kidney	Kidney, pancreas	Pancreas	Kidney	Kidney	Kidney	Kidney	Kidney
Reason for Transplantation	Recurrent HCC (liver cirrhosis child B due to hepatitis C infection)	Idiopathic pulmonary fibrosis	FSGS (primary)	Diabetic nephropathy	Diabetic nephropathy	FSGS (secondary)	Polycystic kidney disease	End-stage renal failure of undetermined origin	Polycystic kidney disease	Diabetic and hypertensive nephropathy
Malignancy	Relapse HCC	SCC	Melanoma	SCC	Squamous cell lung cancer	SCC	SCC	High-grade urothelial carcinoma of the urethra and pulmonary carcinoma	Melanoma	SCC
Metastasis *	Pulmonary, lymphogenic	Pulmonary, lymphogenic	Pulmonary, hepatic, bone, lymphogenic	Nodal, sub- and cutaneous	Pulmonary, nodal	Nodal, local bone infiltration	Hepatic, nodal	Pleural carcinomatosis, nodal	Nodal, hepatic, spleen, bone	Nodal, sub- and cutaneus
TNM-Classification *	ypT2 pN1 M1	N/A	pT2a pN3 M1c	N/A	pT1c pN3 pM1a	N/A	N/A	UNK	pT2a cN3 cM1c	N/A
Tumor mutational burden	UNK	62 Muts/Mb	UNK	48 Muts/Mb	UNK	18 Muts/Mb	UNK	UNK	UNK	39 Muts/Mb
Immunosuppression *	Everolimus	Everolimus, cyclosporine, PDN, ECP	Tacrolimus, PDN	MMF, PDN	Tacrolimus, PDN	Sirolimus, MMF, PDN	Tacrolimus, PDN	Everolimus, PDN	Sirolimus, PDN	Everolimus, MMF, PDN (pulsed after infusion)
ICI (dosage) and its effect pathway	Nivolumab (240 mg) Anti-PD-1	Cemiplimab (350 mg) Anti-PD-1	Ipilimumab (160 mg) Anti-CTLA-4	Cemiplimab (350 mg) Anti-PD-1	Nivolumab (UNK) Anti-PD-1	Cemiplimab (350 mg) Anti-PD-1	Cemiplimab (350 mg) Anti-PD-1	Pembrolizumab (200 mg) Anti-PD-1	Ipilimumab/ nivolumab, followed by nivolumab (240 mg) Anti-CTLA-4, Anti-PD-1	Cemiplimab (350 mg) Anti-PD-1
Therapy	Single dose	2 doses (every 3 weeks)	Cycle 1: 4 doses (every 3 weeks) Cycle 2: 4 doses (every 4 weeks), Cycle 3: 2 doses (every 4 weeks)	4 doses (every 3 weeks)	1 year of treatment (every 2 weeks)	2 doses (every 3 weeks)	4 doses (every 4 weeks)	6 doses (every 3 weeks)	4 doses both drugs (every 3 weeks), followed by only nivolumab (ongoing every 2 weeks)	4 doses (every 3 weeks)
Time since transplantation to initiation of ICI	2 y, 11 m, 28 d	3 y, 2 m, 29 d	6 y, 5 m, 15 d	11 y, 5 m, 24 d	8 y, 5 m, 3 d	6 y, 3 m, 15 d	10 y, 7 m, 21 d	5 y, 2 m, 28 d	8 y, 6 m, 29 d	1 y, 1 m, 5 d
Time since transplantation to cancer diagnosis	2 y, 2 m, 29 d	2 y, 10 m, 23 d	5 y, 6 d	10 y, 7 m, 18 d	6 y, 8 m, 5 d	5 y, 24 d	6 y, 5 d	2 y, 5 m, 2 d	4 y, 6 m	8 m, 29 d

* At the time of initiation of ICI therapy. Abbreviations: ECP, extracorporeal photopheresis; FSGS, focal segmental glomerulosclerosis; HCC, hepatocellular carcinoma; MMF, mycophenolate mofetil; NODAT, New Onset Diabetes After Transplant; PDN, prednisolone; UNK, unknown.

**Table 2:** Primary outcome.

Patient	1	2	3	4	5	6	7	8	9	10
Cancer control	N/A	CR	SD initially, then PD	CR	SD initially, then PD	N/A	PD	PD	CR	PD
Progression-free interval *	N/A	1 m, 8 d	Cycle 1: 6 m, 15 d Cycle 2: 7 m, 9 d Cycle 3: UNK	Ongoing	9 m, 29 d	N/A	2 m, 11 d	4 m, 22 d	Ongoing	2 m, 23 d
Survival/death	Death	Death	Death	Survival	Death	Death	Death	Survival	Survival	Survival
Time period ICI administration to death	25 d	1 m, 8 d	2 y, 7 m, 11 d	N/A	1 y, 8 m, 28 d	1 m, 14 d	3 m 21 d	N/A	N/A	N/A
Time from cancer diagnosis to death	8 m, 27 d	6 m, 14 d	4 y, 10 m, 14 d	N/A	3 y, 5 m, 26 d	1 y, 4 m, 4 d	9 m, 1 d	N/A	N/A	N/A
Cause of death	Spontaneous intracranial mass hemorrhage or coagulopathy due to liver dysfunction, metastatic hemorrhage or PD	Severe respiratory insufficiency (immune-related pneumonitis)	PD	N/A	PD	PD	PD	N/A	N/A	N/A

* At the time of initiation of ICI therapy. Abbreviations: CR, complete response; DD, differential diagnosis; N/A, not applicable; PD, progressive disease; PR, partial response; SD, stable disease; UNK, unknown.

**Table 3:** Secondary outcome.

Patient	1	2	3	4	5	6	7	8	9	10
Graft loss	Yes	Yes	No	Yes	No	Yes	No	Yes	Yes	No
Reason for graft loss	Rejection	Pneumonitis, immune-related	N/A	Rejection	N/A	Rejection	N/A	Thrombotic microangiopathy	Rejection, then nephrectomy	N/A
Graft rejection after starting ICI	After the first dose	N/A	After the first dose	After the first dose	After the first dose	After the second dose	N/A	N/A	After the first dose	N/A
Time period from ICI administration to graft rejection	6 d	N/A	2 m	21 d	17 d	1 m, 2 d	N/A	N/A	20 d	N/A
Clinical findings of graft rejection	Liver enzyme elevation, functional limitation	Severe respiratory partial insufficiency	Creatinine elevation	Creatinine elevation	Hyperglycemia, abdominal pain	Creatinine elevation	N/A	Creatinine elevation	Creatinine elevation	N/A
Biopsy	Cellular rejection	Immune-related Pneumonitis	Interstitial T-cell infiltrates with tubulitis and capillaritis	None taken	None taken	None taken	N/A	Thrombotic microangiopathy, drug-induced (everolimus vs. pembrolizumab)	T-cell-mediated rejection type IIa (Banff Classification)	N/A
Treatment of rejection	PDN, tacrolimus	N/A	PDN	None	PDN	PDN	N/A	N/A	PDN, followed by transplantectomy and dialysis	N/A
Graft response to treatment	No	N/A	Yes	N/A	Yes	No	N/A	N/A	Yes	N/A
Side effects other than graft rejection	No	Immune-related Pneumonitis	Pruritus, skin rash, peripheral dysesthesia	No	No	No	Asthenia, immune-related hepatitis	No other information than possible thrombotic microangiopathy of the graft	Immun-mediated colitis	N/A

Abbreviations: N/A, not applicable; PDN, prednisolone; UNK, unknown.

## Data Availability

The original contributions presented in this study are included in the article. Further inquiries can be directed to the corresponding author.

## Abbreviations

The following abbreviations are used in this manuscript:

CTLA-4	cytotoxic T-lymphocyte-associated protein 4
mTOR	mammalian target of rapamycin
sOTRs	solid organ transplant recipients
STCS	Swiss Transplant Cohort Study
HCC	hepatocellular carcinoma
ICI	immune checkpoint inhibitor
MMF	mycophenolate mofetil
PFS	progression-free survival
SCC	cutaneous squamous cell carcinoma
PD-L1	programmed cell death ligand 1
PD-1	programmed cell death protein 1
CR	complete response
MM	metastatic melanoma
PD	progressive disease
SD	stable disease
